# Interfacial Confinement Effect of Self‐Adsorbed Monolayer Enables Highly Reversible Zn Metal Anodes

**DOI:** 10.1002/advs.202413731

**Published:** 2024-12-31

**Authors:** Yaodong Huo, Shifeng Huang, Zihan Liu, Mengjing Li, Yanjiao Cao, Penghui Tian, Tuotuo Ma, Chenhui Han, Yuliang Gao

**Affiliations:** ^1^ School of Chemistry and Chemical Engineering Inner Mongolia University Hohhot 010021 China; ^2^ School of Physical Science and Technology Inner Mongolia University Hohhot 010021 China

**Keywords:** dendrites, electric double layer, interfacial confinement effect, self‐adsorbed monolayer, Zn metal anodes

## Abstract

The practical applications of aqueous Zn metal batteries are promising, yet still impeded by the corrosion reactions and dendrite growth on the Zn metal anode. Here, a self‐adsorbed monolayer (SAM) is designed to stabilize the Zn metal anode. Theory and experiment results show that the interfacial confinement effect of the SAM, for one thing, greatly suppresses the corrosion reactions through the H_2_O‐poor inner Helmholtz plane because of the steric‐hindrance effect, and for another, alleviates the Zn^2+^ concentration gradient on the anode surface through the Zn^2+^ enrichment behavior and eventually inhibits the dendrite growth. Consequently, the Zn||Cu cell maintains a Coulombic efficiency of 99.3% at 10 mA cm^−2^/1 mAh cm^−2^ for 2000 cycles, and the Zn||Zn cell can stably cycle for 1400 h at 1 mA cm^−2^/1 mAh cm^−2^. Additionally, the NVO||Zn pouch cell shows impressive cycling stability (over 200 cycles) and low gassing behavior at 3 A g^−1^. This work provides a novel perspective for the interface engineering of Zn metal anodes.

## Graphical abstract

A self‐adsorbed monolayer is designed to stabilize the Zn metal anode based on 1‐cyclohexyl‐2‐pyrrolidone molecules. The interfacial confinement effect of the self‐adsorbed monolayer, which refers to the derived steric‐hindrance effect and Zn^2+^ enrichment behavior, can effectively inhibit the corrosion reactions and dendrite growth, and finally endows the Zn metal anode with improved electrochemical performance.

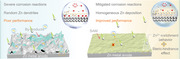

## 1 Introduction

Since the tremendous success of neutral electrolyte‐based rechargeable batteries in 2012, metallic zinc (Zn) has gained widespread recognition as the most viable option for grid‐scale energy storage systems.^[^ ^1^ ^]^ This is associated with the high theoretical capacity (820 mAh g^−1^), suitable redox potential (−0.76 V), plentiful supplies, and inherent safety.^[^ ^2^, ^3^ ^]^ Similar to alkali metal anodes, however, the incompatibility of Zn metal anodes with electrolytes also leads to corrosion reactions and dendrite growth, which seriously impedes the advancement of Zn metal batteries.^[^ ^4^ ^]^

Constructing a stable and reliable electrolyte/anode interface is a crucial strategy to address the issues of corrosion reactions and dendrite growth.^[^ ^5^, ^6^, ^7^ ^]^ Viewed in connection with the lithium‐ion batteries, an elaborate solid electrolyte interphase (SEI) has been considered viable.^[^ ^8^, ^9^, ^10^ ^]^ Unfortunately, the anions in Zn salts with high electrochemical stability prevent the formation of an effective SEI in aqueous Zn metal batteries.^[^ ^11^, ^12^ ^]^ Given that, various functional reagents were employed to regulate the solvation structure of the electrolyte and promote the decomposition of anions to form a stable SEI.^[^ ^13^, ^14^, ^15^, ^16^ ^]^ The fundamental problem lies in the continuous decomposition of functional reagents during cycling, limiting their effectiveness, and also difficulties in the production costs from the high‐concentration electrolytes.^[^ ^17^, ^18^ ^]^ Another promising approach is the construction of artificial interfaces with customizable properties, including inorganic,^[^ ^19^, ^20^, ^21^, ^22^ ^]^ organic,^[^ ^23^, ^24^, ^25^ ^]^ and organic/inorganic protective layers.^[^ ^26^, ^27^, ^28^, ^29^, ^30^ ^]^ Nevertheless, most of these protective layers undergo complicated preparation processes, poor adhesion, and high thickness.^[^ ^31^ ^]^ Obviously, the development of innovative electrolyte/anode interfaces remains essential.

It is worth mentioning that the stability of the electrode/electrolyte interface depends on the microenvironment (active H_2_O and Zn^2+^ concentration) of the anode surface. Specifically, in Zn‐ion battery system, Zn(H_2_O)_6_^2+^ will initially be de‐solvated at the inner electrical double layer (EDL), leaving more active H_2_O on the EDL, which causes serious corrosion reactions.^[^ ^32^ ^]^ Then, the de‐solvated Zn^2+^ will adsorb on the Zn surface and self‐diffuse into the potential sites for nucleation and growth, which is affected by the Zn^2+^ concentration on the anode surface. Ion concentration gradient is the key factor to induce dendrites.^[^ ^33^, ^34^ ^]^ The slow ion mass transfer can not quickly supplement the Zn^2+^ consumed on the anode surface during the electrodeposition process, resulting in the formation of a large Zn^2+^ concentration gradient, eventually inducing the nucleation and growth of dendrites.^[^ ^35^ ^]^ Notably, well‐designed anode microenvironment is a feasible strategy for stabilizing Zn metal anodes, but few reports have examined the intrinsic relationship involved.

In this work, we design a novel self‐adsorbed monolayer (SAM) on the Zn metal anode surface via the 1‐cyclohexyl‐2‐pyrrolidone (CHP) molecules and revealed its interfacial confinement effect. Specifically, the steric‐hindrance effect of the SAM promotes the formation of a H_2_O‐poor inner Helmholtz plane (IHP), which greatly inhibits the corrosion reactions of the Zn metal anode. Meanwhile, the SAM‐induced Zn^2+^ enrichment behavior alleviates the Zn^2+^ concentration gradient on the anode surface, and ultimately suppresses dendrite growth. Benefiting from the interfacial confinement effect of the SAM, which refers to the steric‐hindrance effect and Zn^2+^ enrichment behavior, the electrochemical performance of the Zn metal anode is significantly improved. Notably, the Zn||Cu cell maintains a Coulombic efficiency (CE) of 99.3% for 2000 cycles at 10 mA cm^−2^/1 mAh cm^−2^, the Zn||Zn cell can cycle for 1400 h at 1 mA cm^−2^/1 mAh cm^−2^, and the NVO||Zn pouch cell shows high cycling stability and low gassing behavior at 3 A g^−1^. This work provides a valuable guidance for the interface engineering design of aqueous batteries, and the concept of interfacial confinement effect offers a new perspective for understanding the EDL structure and anode operation mechanism.

## 2 Results and Discussion

### 2.1 Construction and Interfacial Confinement Effect of SAM

**Figure 1:**
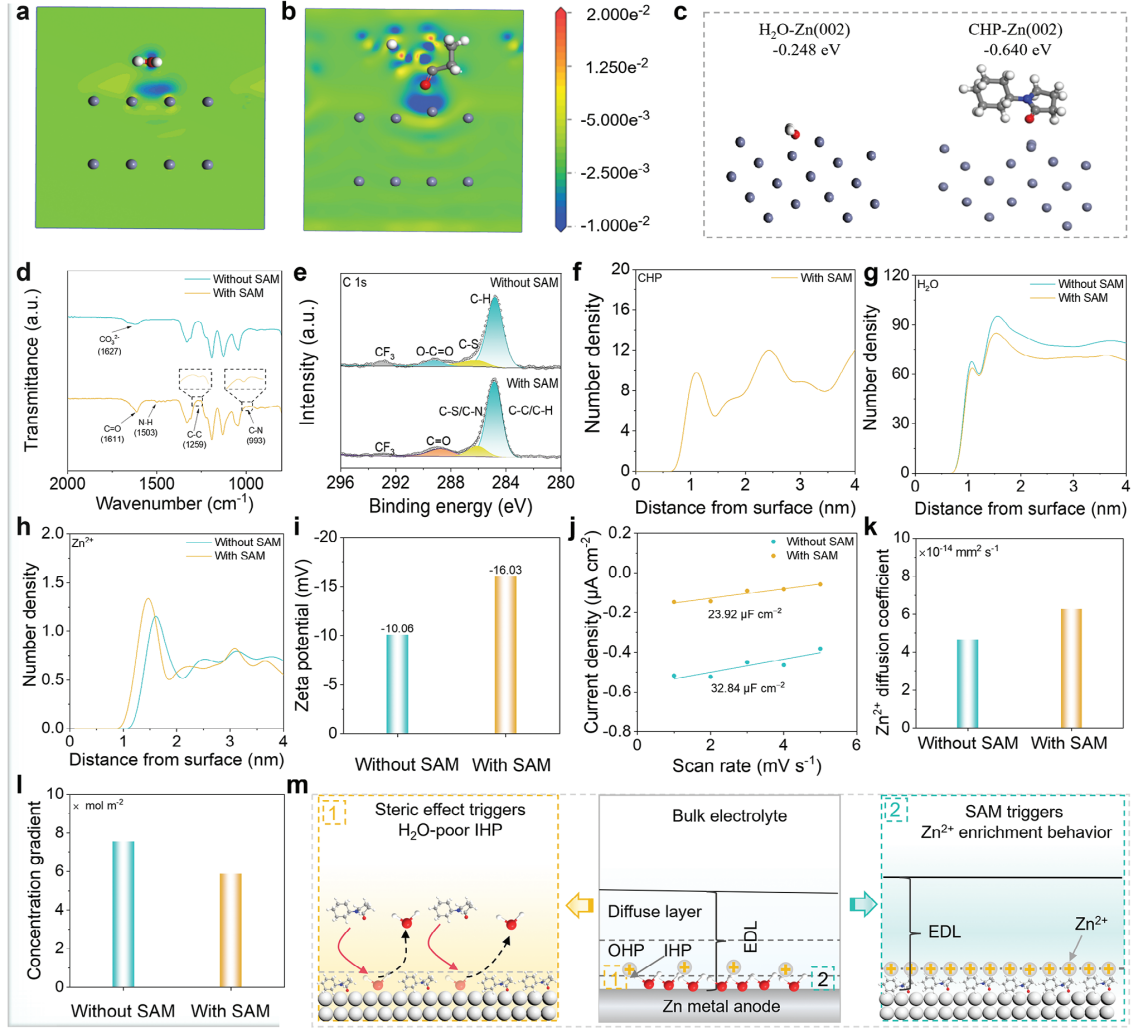
Characterizations of SAM and its interfacial confinement effect. a,b) Charge density difference of H_2_O and CHP on the Zn(002) surface. c) Adsorption energy of H_2_O and CHP on the Zn(002) plane. d) FTIR spectra of the Zn anode surface. e) High‐resolution C 1s XPS spectra of the Zn anode. Distribution density of f) CHP, g) H_2_O, and h) Zn^2+^ along the anode surface. i) Zeta potential of Zn deposits. j) EDL capacitance of Zn deposits. k) Zn^2+^ diffusion coefficients of different anodes. l) Zn^2+^ concentration gradient at the anode surface. m) Schematic illustrations of interfacial confinement effect of SAM.

Unlike conventional artificial protective layers, which often involve complex fabrication processes, the SAM was created by adding CHP additives to the base electrolyte (BE, 1.0 m Zn(TFSI)_2_ + H_2_O). After being mixed with different volume ratios (0.25, 0.5, 1.0, and 2.0%) of CHP, the solutions remain in clear and transparent states (Figure S1, Supporting Information). It means that CHP is uniformly dispersed in the BE, which provides positive conditions for the SAM formation. Meanwhile, Fourier transform infrared (FTIR), Raman, and nuclear magnetic resonance (NMR) spectroscopy were utilized to explore the microstructure of the electrolyte. The results show that the CHP has no significant effect on the solvation structure of the electrolyte, implying that the CHP is more involved in the formation of the SAM (Figures S2–S4, Supporting Information). Furthermore, electrochemical performance tests of Zn||Zn cells at different current densities identified an optimal CHP concentration of 0.5% (Figure S5, Supporting Information). This optimized model electrolyte was then used to explore the electrochemical behaviors of the SAM in detail. Strong interaction between CHP and the Zn substrate is a prerequisite for the formation of the SAM. The electron‐transfer tendency between CHP and Zn(002) is higher than that of H_2_O molecules, confirming a stronger interaction of CHP with the Zn metal anode (**Figure** **1**a,b). This is further validated by density functional theory (DFT) calculations, which show a more negative binding energy for CHP on the Zn(002) surface (Figure 1c).

Besides, FTIR and X‐ray photoelectron spectroscopy (XPS) further confirmed the formation of the SAM. Note that the Zn sheets were immersed in different electrolytes for 12 h before characterization and then rinsed with deionized water and ethanol, respectively. The characteristic peaks of CHP for C─N, C─C, N─H, and C═O appear at 993, 1259, 1503, and 1611 cm^−1^, respectively, indicating that the SAM is successfully formed on the Zn anode surface (Figure 1d). The C 1s spectra of the CHP‐infused Zn foil display intensified signals originating from C═O, C─S/C─N, and C─C/C─H, indicating that CHP is adsorbed on the Zn anode surface (Figure 1e). Meanwhile, compared with the Zn anode without SAM, the F content on the Zn anode surface with SAM is significantly reduced, which is attributed to the inhibition of SAM on corrosion reactions. The O 1s spectra deconvolute three components corresponding to S─O (533.1 eV), C═O (532.3 eV), and Zn─O (530.9 eV),^[^ ^36^ ^]^ which further confirms the existence of the SAM and the strong chemical bonding with the Zn metal anode (Figure S6, Supporting Information). Additionally, the contact angle between the electrolyte and Zn metal anode is reduced from 51.27 to 20.77° after recruiting CHP additives, implying better wettability in the presence of the SAM (Figures S7 and S8, Supporting Information).

The formation of SAM confers a unique interface effect on the Zn metal anode. When the Zn metal anode is immersed in the electrolyte, the excess electrons on the Zn surface cause the ion distribution and H_2_O dipole orientation at the interface to rearrange, forming an EDL.^[^ ^37^ ^]^ Molecular dynamics simulations reveal the interface structure of the Zn metal anode (Figure S9, Supporting Information). For the Zn metal anode without SAM, numerous H_2_O molecules accumulate in the IHP on the anode surface, which is a primary cause of severe corrosion reactions on the bare anode.^[^ ^38^ ^]^ By contrast, the SAM excludes some active H_2_O molecules due to its steric‐hindrance effect, resulting in a decrease in the spatial density of H_2_O molecules on the anode surface and the formation of a H_2_O‐poor IHP (Figure 1f,g). This is beneficial to inhibit the corrosion reactions on the anode surface.^[^ ^39^ ^]^ Meanwhile, the SAM also promotes an increase in Zn^2+^ concentration on the anode surface (Figure 1h). This Zn^2+^ enrichment behavior may be attributed to the attraction of Zn^2+^ by N atoms containing lone pair electrons in CHP (Figure S10, Supporting Information). It is worth noting that the increase of Zn^2+^ concentration helps to reduce the Zn^2+^ concentration gradient on the anode surface, which is crucial for inhibiting dendrite formation and growth.^[^ ^40^ ^]^ Furthermore, compared to Zn deposits without SAM (−16.03 mV), the Zeta potential of the Zn deposits with SAM is −10.06 mV, implying fewer net charges on the Zn anode surface due to the adsorption of Zn^2+^ (Figure 1i).^[^ ^41^ ^]^ In addition, as shown in Figure 1j and Figure S11 (Supporting Information), the capacitance of the electrical double layer (*C*_EDL_) with SAM is much smaller than that of the bare Zn metal anode. According to the equation (*C*_EDL_ =  (ε*A*)/*d*),^[^ ^42^ ^]^ the *C*_EDL_ increases with the thickness of EDL (d) decreasing, indicating a thicker thickness of EDL on the Zn anode surface with SAM with the larger radius of SAM. The potential drop within the EDL with SAM is smaller than that of the anode without SAM due to larger thickness.^[^ ^43^ ^]^ It is well‐known that even a very small potential drop in EDL can lead to a large gap in the electric field due to the thin thickness of EDL.^[^ ^44^ ^]^ This Zn^2+^ enrichment behavior enhances ion transport and also helps to alleviate the ion concentration gradient at the anode surface (Figure 1k,l).

Based on the above analysis, we propose the interfacial confinement effect of the SAM. Confinement, which refers to a defined region, is considered an important strategy for modulating chemical properties in catalysis, energy, and biology.^[^ ^45^, ^46^, ^47^ ^]^ By confining atoms, molecules, and clusters in spaces such as zeolites, carbon nanotubes, and 2D graphene, their fundamental physical and chemical properties can be altered, leading to new chemical reactions and improved performance.^[^ ^48^, ^49^, ^50^ ^]^ In contrast to closed spaces, the confinement effect on open surfaces/interfaces also has an important impact on catalysis, while batteries receive less attention. Apparently, the construction of SAM responds to the EDL region of the anode surface (Figure 1m). On the one hand, the steric‐hindrance effect of the SAM promotes the formation of the desired H_2_O‐poor IHP within the EDL on the anode surface, which contributes to the inhibition of the corrosion reactions on the anode surface. On the other hand, the Zn^2+^ enrichment behavior of the SAM helps to alleviate the Zn^2+^ concentration gradient on the anode surface and inhibit dendrite growth. Here, the steric‐hindrance effect, Zn^2+^ enrichment behavior, and the related electrochemical behavior induced are considered the interfacial confinement effect of SAM.

### 2.2 Anti‐Corrosion Behavior

**Figure 2:**
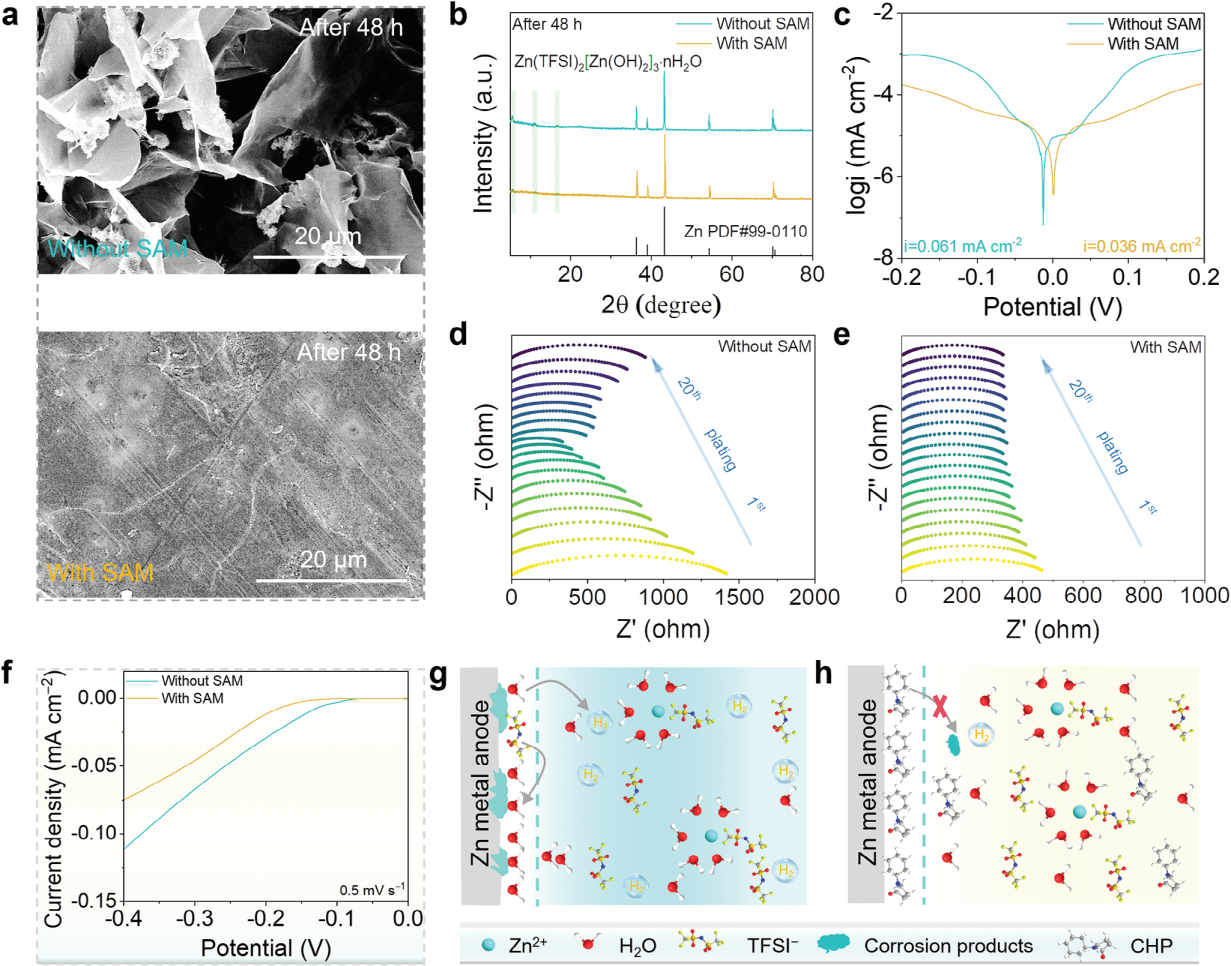
Anti‐corrosion reactions of SAM on anode surface. a) SEM images and b) XRD patterns of Zn metal anodes without and with SAM after 48 h immersion in electrolyte. c) Tafel curves of Zn metal anodes without and with SAM. In situ EIS curves of Zn||Zn cells d) without and e) with SAM. f) LSV curves of Zn metal anodes without and with SAM. Schematic illustrations of corrosion reactions on anode surface g) without and h) with SAM.

The H_2_O‐poor IHP induced by the steric‐hindrance effect of SAM helps to inhibit the corrosion reactions on the anode surface. As the scanning electron microscope (SEM) images shown in **Figure** **2**a, the anode surface without SAM is severely corroded after soaking in the electrolyte for 48 h, forming massive flake‐like products. As a comparison, the Zn metal anode with SAM presents a smooth surface. XRD patterns display that the anode surface without SAM shows a by‐product of Zn(TFSI)_2_[Zn(OH)_2_]_3_·nH_2_O, while there are no residual products on the anode surface with SAM (Figure 2b). Obviously, the construction of SAM can effectively inhibit the corrosion reactions on the anode surface. Tafel curves in Figure 2c reveal that the Zn metal anode with SAM presents a lower corrosion current (0.036 mA cm^−2^) compared to the anode without SAM (0.061 mA cm^−2^), which is attributed to the steric‐hindrance effect of SAM that reduces the content of reactive H_2_O within the IHP, mitigating possible corrosion reactions. The results are further confirmed by the electrochemical impedance spectroscopy (EIS) of Zn||Zn cells after standing for different times, in which the cells with SAM present smaller electrochemical impedance values (Figure S12, Supporting Information). To further investigate the effectiveness of SAM in inhibiting the corrosion reactions on the anode, we evaluated the changes through in situ EIS of the Zn||Zn cells during charging and discharging. According to Figure 2d, the electrochemical impedance value of the Zn||Zn cell without SAM shows a decreasing and then increasing trend during the charging and discharging process from the 1^st^ to the 20^th^ cycle. This happens because of the cell activation at the pre‐cycle stage and the severe corrosion behavior at the post‐cycle stage. In contrast, the cell with SAM presents a smaller electrochemical impedance value and remains stable during the charging and discharging process (Figure 2e). Such a noticeable comparison shows that SAM has excellent anti‐corrosion performance due to H_2_O‐poor IHP induced by the steric‐hindrance effect.

Furthermore, Figure 2f compares the linear sweep voltammetry (LSV) curves of different Zn metal anodes. Compared with the anode without SAM, the anode with SAM has a more negative hydrogen evolution overpotential. More importantly, the inhibition of SAM on the corrosion reactions of the anode surface endows the battery with excellent self‐discharge performance (Figure S13, Supporting Information). Specifically, the capacity retention rates of NVO||Zn battery are of 75.3% without SAM and 83.5% with SAM after a 48 h self‐discharge test. The inhibition mechanism of SAM on the corrosion reactions of the Zn metal anode can be described as follows: For the anode without SAM (Figure 2g), H_2_O molecules in the electrolyte preferentially occupy IHP due to its solvation structure and fast diffusion kinetics, thus forming a H_2_O‐rich EDL structure. During the desolvation process, massive active H_2_O molecules are released, and the hydrogen evolution reaction (HER) occurs on the anode surface. Then the HER accelerates the corrosion rate and produces deadly OH^−^ in IHP, resulting in the formation of insulating and uneven by‐products, which eventually continue to corrode the Zn metal anode and cause the electrolyte to dry up. Unlike this, the steric‐hindrance effect of the SAM promotes the formation of a H_2_O‐poor IHP on the anode surface (Figure 2h). For one thing, the H_2_O‐poor IHP inhibits the HER of active H_2_O during the desolvation process. And for another, the SAM physically blocks the corrosion reactions between the electrolyte and the active Zn metal, endowing the Zn metal anode with excellent anti‐corrosion performance.

### 2.3 Zn Electrodeposition Behavior

**Figure 3:**
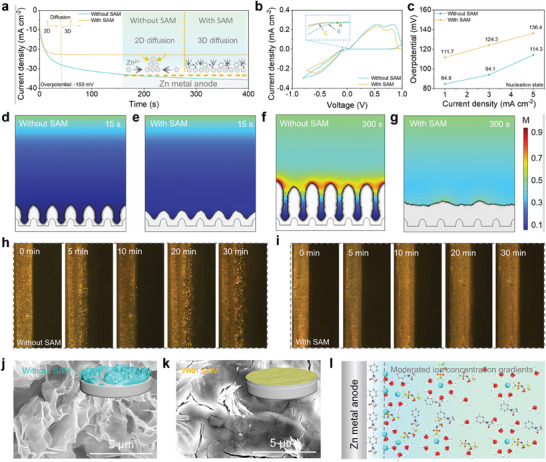
Zn electrodeposition behavior based on SAM. a) Chronoamperometry curves of Zn||Zn cells at a constant overpotential of −150 mV. b) CV curves of Zn metal anode. c) Comparison of nucleation overpotential of Zn metal at different current densities. Simulated ion concentration distributions during the process of Zn deposition on the Zn metal anodes d,f) without and e,g) with SAM. In situ optical microscopy visualization of Zn deposition on Zn metal anodes h) without and i) with SAM at 10 mA cm^−2^. SEM images of Zn deposits with 1 mAh cm^−2^ (1 mA cm^−2^) capacity on anode surfaces j) without and k) with SAM (The inserts are corresponding morphology diagrams). l) Schematic illustration of SAM on Zn electrodeposition behavior.

The Zn^2+^ enrichment behavior induced by the SAM plays a crucial role in regulating the Zn electrodeposition behavior. In **Figure** **3**a, the current response after 400 s at an overpotential of ‐150 mV is shown, representing the fully discharged state of the Zn metal anode. Without SAM, Zn plating on the anode surface shows a continuously increasing current density with typical 2D diffusion curves. This kind of diffusion behavior is more likely to result in uneven nucleation, leading to the formation of Zn dendrites. Instead, a stable and constant 3D diffusion process occurred on the anode surface with SAM after a short nucleation state for 40 s. This indicates that SAM‐induced Zn^2+^ enrichment behavior inhibits 2D diffusion of Zn^2+^, promotes uniform nucleation of Zn^2+^, and inhibits the formation of Zn dendrites. The nucleation overpotentials (η_BA_ and η_CA_) were determined by the cyclic voltammetry (CV) curves, showing that the Zn metal anode with SAM has a higher nucleation overpotential, indicating the SAM contributes to achieving a dense and uniform deposition morphology (Figure 3b). The voltage‐capacity curves of Zn||Cu cells at different current densities further prove this result (Figure 3c; Figure S14, Supporting Information). The nucleation overpotential of Zn metal rises with increasing current density. Meanwhile, compared with the Zn metal anode without SAM, the anode with SAM exhibits a larger nucleation overpotential.

COMSOL simulations provide comprehensive insights into the effect of SAM on Zn electrodeposition behavior (Figure 3d–g; Figure S15, Supporting Information). The protrusions on the anode surface facilitate ion aggregation, leading to preferential reduction of ions. Over time, these protrusions transform into dendrites. Fortunately, the introduction of SAM significantly reduces the Zn^2+^ concentration gradient at the protrusions, promoting uniform deposition of Zn^2+^. Even after 300 s of deposition, a uniform dendrite‐free morphology is maintained. On this basis, we also used an in situ optical microscope to record the Zn electroplating process. Side‐view images reveal that some protuberances appear randomly on the Zn foil surface without SAM after plating of 5 min, which gradually evolve into conspicuous Zn dendrites (Figure 3h). In stark contrast for the Zn metal anode with SAM, uniform deposition of Zn without discernible protuberances during the whole plating process of 30 min except for the continuously thickened Zn foil occurs (Figure 3i). SEM images provide further evidence that SAM modulates Zn electrodeposition. The Zn surface without SAM is covered by massive, thin, and sharp flakes in ruleless orientations, which leads to a loose and messy structure (Figure 3j), whereas the SAM readily leads to a densely stacked and compact Zn surface (Figure 3k). Even subjected to elevated current density, the Zn metal anodes with SAM can still exhibit smooth and compact surface morphology, manifesting that the uniform Zn deposition can be realized via SAM (Figure S16, Supporting Information). The primary reason lies in that when in the absence of SAM, the huge electric field gradient in the EDL renders concentrated Zn^2+^ flux and fast Zn^2+^ transfer, which triggers uneven Zn deposition and the rapid formation of Zn dendrites (Figure S17, Supporting Information). In contrast, the SAM‐induced Zn^2+^ enrichment behavior enhances the Zn^2+^ concentration on the anode surface and alleviates the Zn^2+^ concentration gradient near the anode. This effectively regulates the Zn electrodeposition and ultimately achieves dendrite suppression (Figure 3l).

### 2.4 Reversibility of Zn Plating/Stripping

**Figure 4:**
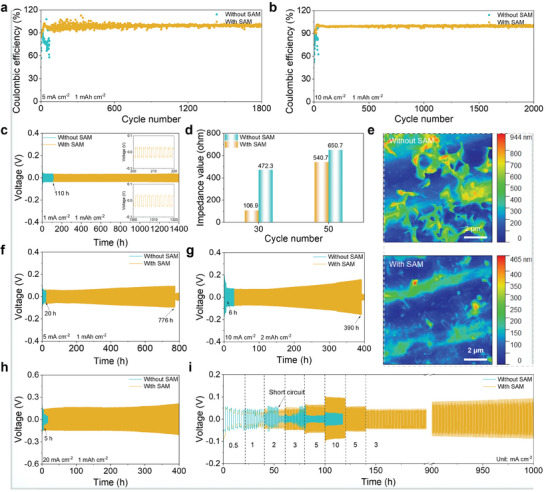
Plating/stripping performance of SAM. CE of Zn||Cu cells at a) 5 mA cm^−2^/1 mAh cm^−2^ and b) 10 mA cm^−2^/1 mAh cm^−2^. c) Cycling performance of Zn||Zn cells at 1 mA cm^−2^/1 mAh cm^−2^ (the inserts are local voltage‐time curves). d) Comparison of electrochemical impedance of Zn||Zn cells without and with SAM after cycling at 1 mA cm^−2^/1 mAh cm^−2^. e) AFM images of Zn metal anodes without and with SAM after 50 cycles in Zn||Zn cells at 1 mA cm^−2^/1 mAh cm^−2^. Cycling performance of Zn||Zn cells at f) 5 mA cm^−2^/1 mAh cm^−2^, g) 10 mA cm^−2^/2 mAh cm^−2^, and h) 20 mA cm^−2^/1 mAh cm^−2^. i) Rate performance of Zn||Zn cells.

To evaluate the interfacial confinement effect of the SAM on the reversibility of Zn plating/stripping, the Zn||Cu cells were first assembled. CE is a crucial metric for evaluating the plating/stripping reversibility of Zn metal anodes. The CE of Zn plating/stripping on Cu foil are presented in Figures S18 and S19 (Supporting Information). In a cell without SAM, the CE fluctuates significantly during charging and discharging, a result of intense corrosion reactions and dendrite growth. In contrast, the cell with SAM maintains a stable CE, remaining steady for over 310 and 1600 cycles at 1 and 3 mA cm^−2^, respectively. Even at an increased current density of 5 mA cm^−2^ (**Figure** **4**a), the cell still maintains a stable CE of 1800 cycles, whereas the cell without SAM occurs erratically. This impressive performance is attributed to the effective suppression of corrosion reactions and dendrite growth by the SAM. When further increased to 10 mA cm^−2^ (Figure 4b), the cell with SAM maintains a CE of 99.3% after 2000 cycles. The improved CE underscores that the SAM significantly enhances the plating/stripping reversibility of the Zn metal anode, which is essential for enhancing the cycle life of Zn metal batteries.

Zn||Zn cells were also employed to evaluate the reversibility of Zn plating/stripping. As depicted in Figure 4c, at 1 mA cm^−2^, the Zn||Zn cell without SAM experiences a short circuit fault of only 110 h. Conversely, the cell with SAM operates normally for more than 1400 h, which is superior to the conventional protective layer and electrolyte modification strategies reported in the literature (Table S1, Supporting Information). This remarkable performance lies in, on the one hand, the suppression of parasitic reactions within the cell by SAM, as confirmed in Figure 4d and Figure S20 (Supporting Information) that the Zn||Zn cell consistently maintains a lower electrochemical impedance during the cycling process. On the other hand, this is also attributed to the inhibition of Zn dendrites by SAM during charging and discharging, as shown in 2D atomic force microscope (AFM) images (Figure 4e) and SEM images (Figure S21, Supporting Information). Compared with the rough and loose dendrite morphology of the bare Zn metal anode, the SAM endows the anode with a dense and flat dendrite‐free morphology. At 3 mA cm^−2^, the SAM also endows a cell lifetime of more than 1000 h (Figure S22, Supporting Information). Unfortunately, the cell without SAM can only run for 48 h. Increasing the current density to 5 mA cm^−2^ (Figure 4f), the Zn||Zn cell with SAM has a long lifespan of 776 h, compared to only 20 h without SAM. Additionally, when the deposition capacity increases to 2 mAh cm^−2^ (Figure S23, Supporting Information), instead of the cell without SAM cycles only 14 h, the Zn||Zn cell with SAM cycles stably for over 400 h at 5 mA cm^−2^. At a higher current density of 10 mA cm^−2^ (Figure S24, Supporting Information), the Zn||Zn cell with SAM obtains an excellent lifetime of 600 h relative to only 10 h without SAM. Further increasing to 2 mAh cm^−2^ (Figure 4g), the Zn||Zn cell with SAM maintains excellent cycling stability for over 390 h at 10 mA cm^−2^, while the cell without SAM failed after 6 h, confirming the positive effect of SAM on the stability of Zn plating/stripping. Surprisingly, even at a demanding current density of 20 mA cm^−2^, the Zn||Zn cell with SAM are able to operate properly and cycle for over 400 and 234 h at deposition capacities of 1 and 2 mAh cm^−2^, respectively, while the cell without SAM exhibits poor performance (Figure 4h; Figure S25, Supporting Information). The rate performance of the Zn||Zn cell further confirms the stability of SAM for Zn plating/stripping. As shown in Figure 4i, as the current density increases from 0.5 to 10 mA cm^−2^, the voltage polarization of the cell with SAM increases steadily, while the cell without SAM suddenly decreases suddenly at 2 mA cm^−2^ due to an internal short circuit.

### 2.5 Service Performance of SAM

**Figure 5:**
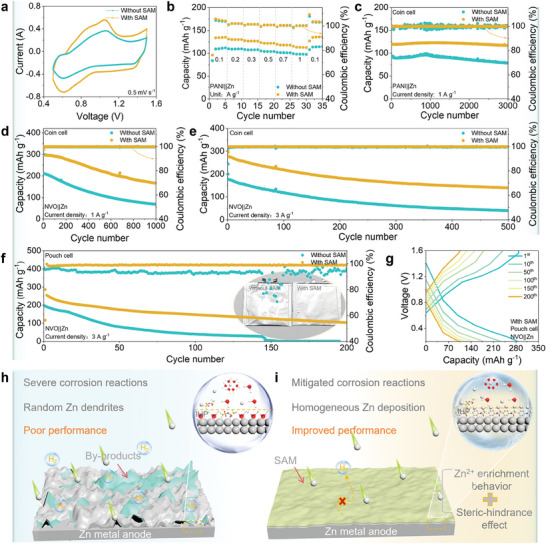
Service performance of SAM in full cell. a) CV curves of PANI||Zn full cell at a scan rate of 0.5 mV s^−1^. b) Rate performance of PANI||Zn full cell. c) Cycling performance of PANI||Zn full cell at 1 A g^−1^. Cycling performance of NVO||Zn full cells at d) 1 and e) 3 A g^−1^. f) Cycling performance of NVO||Zn pouch cell at 3 A g^−1^. g) Typical charge/discharge profiles of NVO||Zn pouch cell with SAM. Schematic illustration of Zn metal anodes h) without and i) with SAM.

To assess the actual service behavior of SAM, the full cells were assembled using polyaniline (PANI) as the cathode and Zn foil as the anode. The CV curves of PANI||Zn cell with SAM, as shown in **Figure** **5**a, exhibit a smaller voltage polarization and a higher peak current density, indicating good reversibility and electrochemical reactivity. Consequently, from 0.1 to 1 A g^−1^, the cell shows an excellent reversible capacity (Figure 5b). Importantly, the value returns to starting points upon reversing the current density, evidencing that the SAM conferred excellent reversibility to the full cell. Concurrently, the corresponding PANI||Zn cell with SAM also exhibits much better cycling stability than that without SAM. In detail, the cell with SAM has a capacity of 117.1 mAh g^−1^ at 1 A g^−1^ after 3000 cycles, as illustrated in Figure 5c, compared to 77.6 mAh g^−1^ without SAM. At 3 A g^−1^, the cell with SAM can still maintain a capacity retention rate of 95.3% (107.9 mAh g^−1^) after 2000 cycles, in contrast to only retaining 65.7% of its capacity (64.2 mAh g^−1^) without SAM (Figure S26, Supporting Information). The improved performance is attributed to the suppression of corrosion reactions and dendrites by SAM, as confirmed by the smaller electrochemical impedance value (Figure S27, Supporting Information).

Furthermore, NH_4_V_4_O_10_ (NVO)||Zn cells were assembled to assess the service behavior of the SAM. With the assistance of the SAM, the cell exhibits a more attractive rate and reversible performance (Figure S28, Supporting Information). Additionally, the SAM contributed to enhanced stable cycling performance at 1 A g^−1^ (Figure 5d). And even at a higher current density of 3 A g^−1^, the cell with SAM maintains 85.7% capacity retention after 500 cycles (Figure 5e). In contrast, the cell without SAM exhibits unsatisfactory behavior with a significantly lower and rapidly decreasing capacity of 19.6%, owing to the large polarization caused by corrosion reactions and dendrite growth (Figure S29, Supporting Information). Subsequently, the pouch cell with a sandwich structure of cathode‐separator‐anode, is further employed to evaluate the service behavior of the SAM. Consistent with the coin cell, the cycling performance of the pouch cell with SAM is significantly improved. Specifically, the pouch cell with SAM has a capacity of 98.9 mAh g^−1^ after 200 cycles at 3 A g^−1^ and exhibits a low gassing behavior (Figure 5f). In contrast, the pouch cell without SAM exhibits fast capacity decay (only 7.4 mAh g^−1^ after 146 cycles) and a noticeable gassing behavior. The observed performance disparity can be attributed to the inhibitory effect of the SAM on severe corrosion reactions and dendrite growth, resulting in reduced cell polarization, as evidenced by the representative charge/discharge curves (Figure 5g; Figure S30, Supporting Information).

Based on the aforementioned analyses, we propose the mechanism of SAM on the Zn metal anode. Specifically, in the absence of SAM (Figure 5h), the H_2_O‐rich IHP in the EDL continuously corrodes the exposed Zn metal, resulting in HER and derivative by‐products at the interface. Meanwhile, a large Zn^2+^ gradient on the anode surface during electrodeposition triggers the formation and growth of dendrites. Severe corrosion reactions and random dendrite growth result in a Zn metal anode with poor performance. In contrast, for the anode with SAM (Figure 5i), the H_2_O‐poor IHP was constructed by the steric‐hindrance effect caused by SAM, which greatly mitigates the corrosion reactions on the anode surface. Meanwhile, the SAM‐induced Zn^2+^ enrichment behavior enhances the Zn^2+^ concentration on the anode surface and alleviates the Zn^2+^ concentration gradient near the anode, which effectively homogenizes the Zn electrodeposition and achieves dendrite suppression. Ultimately, the interfacial confinement effect of SAM endows the Zn metal anode with improved performance.

## 3 Conclusion

In summary, a SAM was designed to stabilize the Zn metal anode based on the strong interaction between CHP and Zn metal. The steric‐hindrance effect of the SAM can exclude adsorbed H_2_O molecules in the IHP and promote the formation of a H_2_O‐poor IHP, which inhibits corrosion reactions at the anode. Meanwhile, the SAM‐induced Zn^2+^ enrichment behavior alleviates the Zn^2+^ concentration gradient on the anode surface, ultimately inhibiting Zn dendrite formation. Benefiting from the interfacial confinement effect of SAM, the Zn metal anode obtains excellent electrochemical performance. Specifically, the Zn||Cu cell with SAM maintains a CE of 99.3% at 1 mA cm^−2^/1 mAh cm^−2^ for 2000 cycles, whereas the cell without SAM exhibits spurious CE and fails to operate properly. Further, the Zn||Zn cell with SAM can stably cycle over 1400 h at 1 mA cm^−2^/1 mAh cm^−2^, ≈13 times the lifetime of the cell without SAM (110 h). Moreover, the sandwich‐structured NVO||Zn pouch cell with SAM can cycle at 3 A g^−1^ for more than 200 cycles (98.9 mAh g^−1^), exhibiting a low gassing behavior. In contrast, the pouch cell without SAM shows a fast decay behavior (only 7.4 mAh g^−1^ after 146 cycles) and a noticeable gassing behavior. This work enriches the understanding of interface engineering and provides new insights into designing facile and feasible protective layers for Zn metal anodes.

## Conflict of Interest

The authors declare no conflict of interest.

## Supporting information



Supporting Information

## Data Availability

The data that support the findings of this study are available from the corresponding author upon reasonable request.
