# Supplementary material for: Interfacial Confinement Effect of Self‐Adsorbed Monolayer Enables Highly Reversible Zn Metal Anodes
Source: Adv Sci (Weinh). 2024 Dec 31;12(8):2413731. doi: 10.1002/advs.202413731 (PMC11848536; doi:10.1002/advs.202413731)
Supplement: Supplementary file 1 — Supporting Information [file ADVS-12-2413731-s001.docx]

**Supporting information**

**Interfacial Confinement Effect of Self-adsorbed Monolayer Enables Highly Reversible Zn Metal Anodes**

*Yaodong Huo^a^, Shifeng Huang^a^, Zihan Liu^a^, Mengjing Li^a^, Yanjiao Cao^a^, Penghui Tian^a^, Tuotuo Ma^b,*^, Chenhui Han^a^, Yuliang Gao^a,*^*

Y. Huo, S. Huang, Z. Liu, M. Li, Y. Cao, P. Tian, C. Han, Y. Gao

^a^School of Chemistry and Chemical Engineering, Inner Mongolia University, Hohhot 010021, China
E-mail: ylgao@imu.edu.cn

1. Ma

^b^School of Physical Science and Technology, Inner Mongolia University, Hohhot 010021, China

E-mail: ttma@imu.edu.cn

**Method**

**Materials**

Zn(TFSI)_2_·2H_2_O (99.9%) was purchased from Beijing MREDA Technology Co., LTD. 1-Cyclohexyl-2-pyrrolidone (CHP, 98%), H_2_C_2_O_4_·2H_2_O (≥99.5%), and NH_4_VO_3_ (99%) were purchased from Shanghai Aladdin Biochemical Technology Co., LTD. Polyaniline (PANI, 98%) was purchased from Shanghai Maclin Biochemical Technology Co., LTD. Carbon paper (HCP020N), polyvinylidene fluoride (PVDF), polytetrafluoroethylene (PTFE), Super C 45, and Ketjen black were purchased from Dongguan Kelud Innovation Technology Co., LTD. Zn foil (30 µm, >99%) and Ti foil (30 µm, >99%) were purchased from Guangzhou Kehua Scientific Instrument Trading Co., LTD.

**Preparation of self-adsorbing monolayer**

The preparation of the self-adsorbed monolayer was achieved by configuring a functional electrolyte containing CHP. Note that the CHP added to the base electrolyte (BE) spontaneously adsorbs on the anode surface to form a self-adsorbed monolayer. Here the functional electrolyte was prepared by dissolving 1.0 M Zn(TFSI)_2_·2H_2_O in deionized water with different volume ratios of CHP from 0, 0.25, 0.5, 1.0, and 2.0%, expressed as BE, BE+0.25CHP, BE+0.5CHP, BE+1.0CHP, and BE+2.0CHP.

**Synthesis of NH_4_V_4_O_10_ cathode material**

NH_4_V_4_O_10_ (NVO) was synthesized by the hydrothermal method. As described below, 0.6317 g NH_4_VO_3_ was dissolved in 35 mL of deionized water and stirred at room temperature for 30 min, and then 0.7514 g H_2_C_2_O_4_·2H_2_O was added and stirred for another 1 h until it turned a light green color. The above-mixed solution was transferred to a 50 mL PTFE-lined autoclave that was kept at 120 °C for 2 h. Then the NVO powder was obtained by centrifugation with deionized water and anhydrous ethanol three times and vacuum drying for 12 h.

**Preparation of NVO or PANI cathode sheets**

The prepared NVO, super P, and PVDF were ground in an agate mortar in a mass ratio of 7:2:1 for 30 min, and then dispersed in 1-methyl-2-pyrrolidone (NMP) to stir for 12 h. Finally, the slurry was uniformly coated on the Ti foil and dried overnight at 60 °C to obtain NVO cathode sheets. PANI cathode sheets were prepared by grinding commercial PANI, Ketjen black, and PTFE in a mass ratio of 5:4:1 for 2 h, then pressing the mixed powder onto carbon paper. Note that the active material loads of NVO and PANI were about 1.0 mg cm^−2^.

**Physicochemical characterizations**

The morphology of the samples were characterized by scanning electron microscopy (SEM, Hitachi S-4800). X-ray diffraction (XRD) patterns were recorded with a PANalytical microdiffractometer (Empyrean) using Cu-Kα_1_ radiation (λ = 1.5406 Å) from 5° to 80°. The microstructure morphology of the Zn metal anode was measured using an atomic force microscope (AFM, BRUKER Dimension Icon). Fourier transform infrared spectroscopy (FTIR, Bruker Tensor 27), Raman spectra (UHTS 600 SMFC VIS, 532 nm), and nuclear magnetic resonance (NMR, Bruker, 600 MHz) were used to characterize the microstructures of the electrolytes. X-ray photoelectron spectroscopy (XPS, Thermo Scientific Escalab 250Xi) was performed on a spectrometer to analyze the chemical compositions. The contact angles between the different electrolytes and Zn foils were recorded on a contact angle meter (Dataphysicals, OCA20). The Zeta potential was performed on a potential analyzer (Brookhaven 90 Plus).

**Electrochemical measurements**

2032-type coin cells (Zn||Cu, Zn||Zn, PANI||Zn and NVO||Zn) were assembled in the ambient environment using a glass fiber separator (Whatman, GF/D). The volume of electrolyte added to each coin cell is 120 μL, and the pouch cell is 2 mL. The NVO||Zn pouch cell used in the experiment showed a sandwich structure (cathode-separator-anode) with a size of 5 cm × 4 cm. All full cells were activated for 2 cycles at 0.1 A g^−1^ before the long-term cycling test.

All of the cells were monitored using a NEWARE test machine (MIHW-200-160CH-B) at 25 °C. Chronoamperometry, linear sweep voltammetry (LSV) and electrochemical impedance spectroscopy (EIS; frequency range: 0.01 Hz ~ 100 kHz) were conducted on an electrochemical workstation (CHI760E, Chenhua). For the PANI||Zn full cell, the voltage ranges of CV tests were 0.5 to 1.5 V. In situ optical images for the Zn deposition process were obtained by a high-resolution optical microscope (YM710R, YUECSOPE). The Zn||Zn symmetrical cells were stood for 6 h before the in-situ EIS tests. The self-discharge performance was evaluated based on NVO||Zn full cell using a previously reported method^[1]^.

**Theoretical calculations and simulation details**

Quantum chemistry (QC) calculations are performed with Gaussian16 software to investigate the interaction between ions or molecules. B3LYP functional is used as it is robust for both main group elements and transition elements. GD3(0) dispersion correction is used to improve the precision of weak interactions. The 6-311+G (d, p) basis set was used for C, H, O, S, F, and N atoms, while the Lanl2TZ basis set is used for Zn atoms. The restrained electrostatic potential (RESP) atom charges and electrostatic potential (ESP) are calculated through Multiwfn software. The force field parameters of Zn^2+^ ions and the TIP3P water model are obtained with the Amber99SB force field. The GAFF force field parameters of CHP and TFSI^-^ molecules are generated with the Acpype program. RESP atom charges were used to describe electrostatic interactions. The atomic charges of all ions were multiplied by a scale factor of 0.70 to correct the polarization effect of ions.

Zn electrodeposition simulations were studied by COMSOL Multiphysics software. According to the Butler-Volmer equation (Eq. 1), the local current density can be calculated as follows:

| $i_{\mathrm{loc}}=i_{0}$(exp($\frac{\alpha_{a}F\eta}{RT}$)*−*exp($\frac{{-\alpha}_{c}F\eta}{RT}$)) | (1) |
| --- | --- |

Where *i*_loc_ is the current density of the electrode, *i*_0_ represents the exchange current density, α_a_ represents the charge transfer coefficient in the anode direction, α_c_ represents the charge transfer coefficient in the cathode direction, F is the Faraday constant, η is the activation overpotential, R represents the ideal gas constant, and T is the temperature in Kelvin.

The diffusion and electric field migration equation (Eq. 2) were used to study the ion migration:

| $N_{\mathrm{Zn}}={-D}_{\mathrm{Zn}}{\nabla c}_{\mathrm{Zn}}-Z_{\mathrm{Zn}}\mu_{m, Zn}{Fc}_{\mathrm{Zn}}{\nabla\Phi}_{l}$ | (2) |
| --- | --- |

Where $N_{\mathrm{Zn}}$ is the Zn^2+^ diffusion flux, *D*_Zn_ represents the Zn^2+^ diffusion coefficient, ${\nabla c}_{\mathrm{Zn}}$ is the Zn^2+^ concentration gradient, $\mu_{m, Zn}$ represents the Mobility of Zn^2+^, $Z_{\mathrm{Zn}}$ is the Zn^2+^ band charge and ${\nabla\Phi}_{l}$ represents the electric potential in solution.


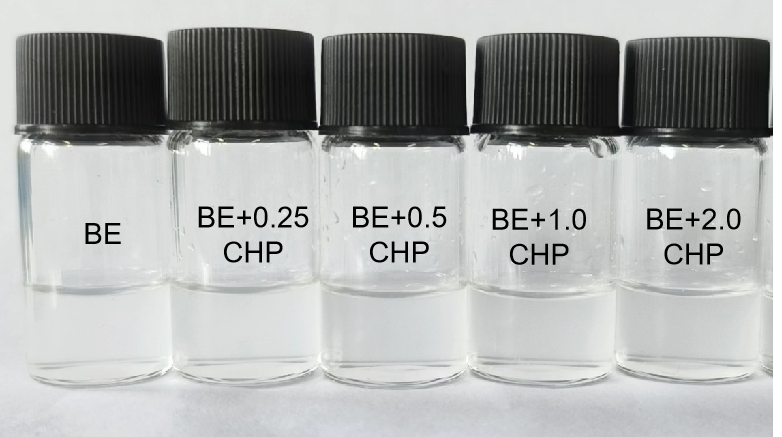


**Figure S1.** Optical photos of different electrolytes.


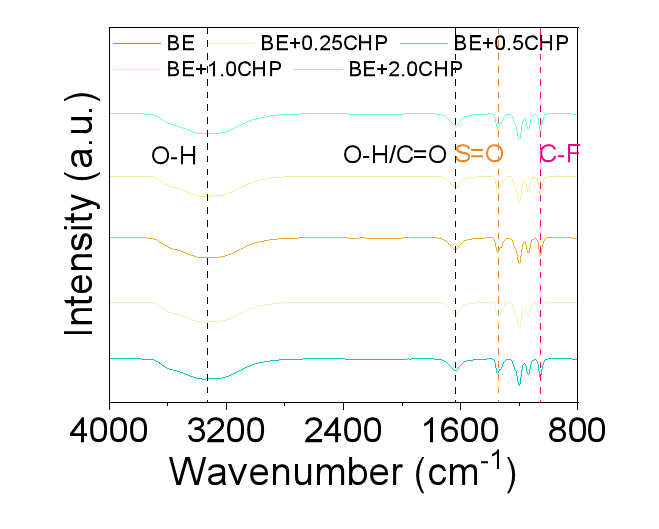


**Figure S2.** FTIR spectroscopy of different electrolytes.


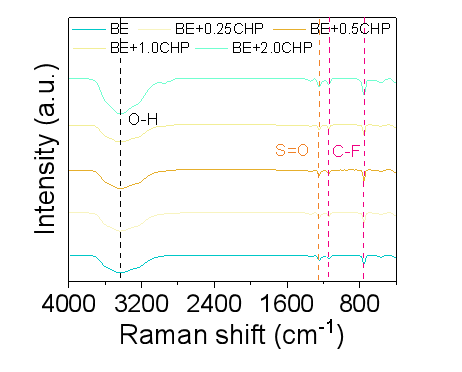


**Figure S3.** Raman spectra of different electrolytes.


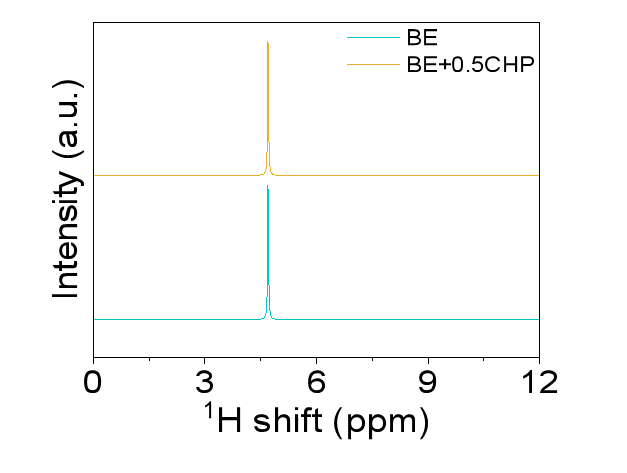


**Figure S4.** ^1^HNMR spectroscopy of different electrolytes.


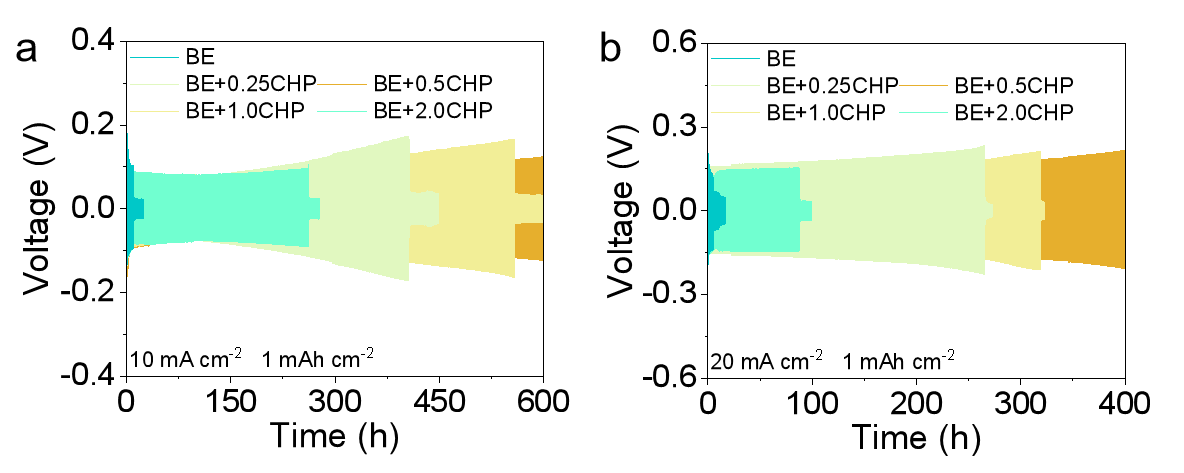


**Figure S5.** Cycling performance of Zn||Zn cells with different electrolytes at (a) 10 mA cm^–2^/1 mAh cm^–2^ and (b) 20 mA cm^–2^/1 mAh cm^–2^.


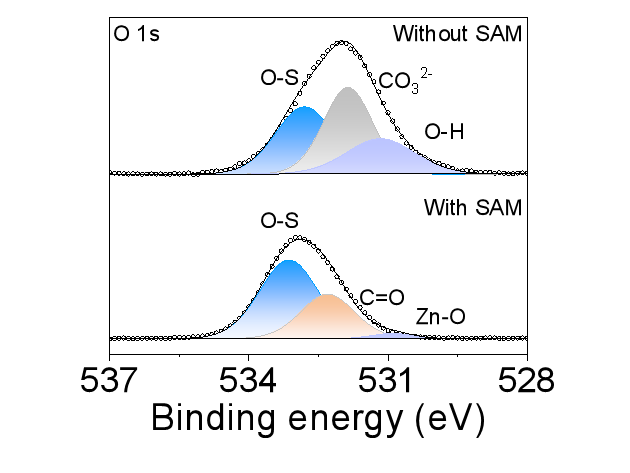


**Figure S6.** High-resolution O 1s XPS spectra of different Zn anodes.


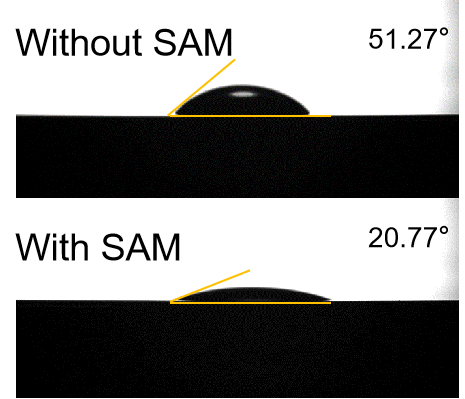


**Figure S7.** Contact angles of Zn anodes without and with SAM.


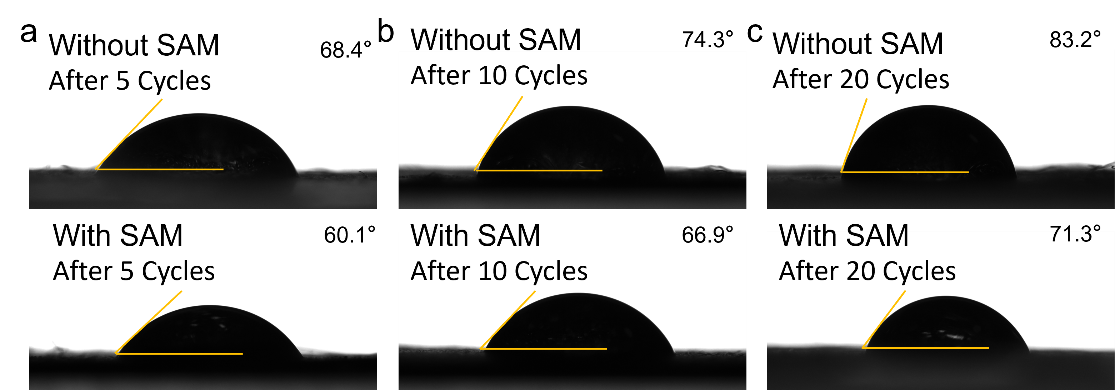


**Figure S8.** Contact angles of Zn anodes without and with SAM after 20 cycles at 1 mA cm^–2^/1 mAh cm^–2^.


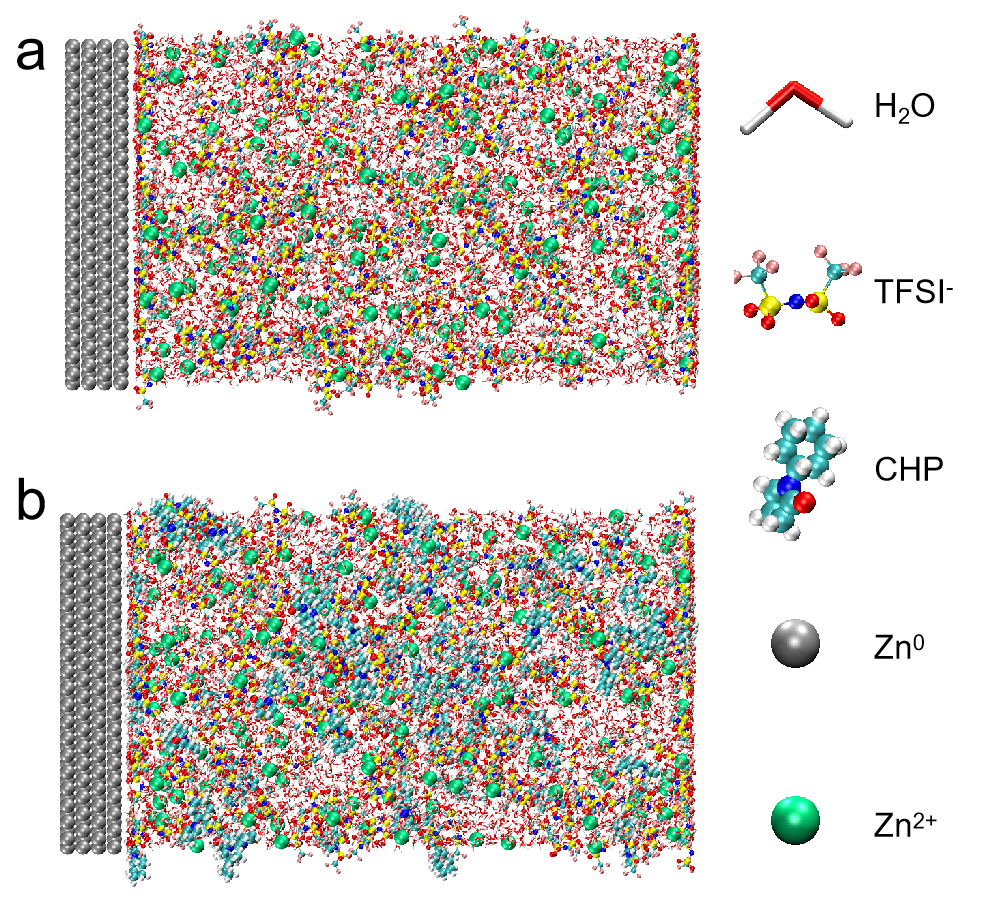


**Figure S9.** Snapshots of simulated structures of the electrolytes (a) without and (b) with CHP.


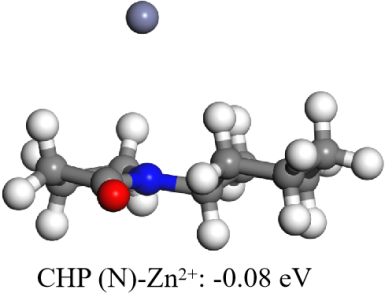


Figure S10. The binding energy between CHP (N) and Zn^2+^.


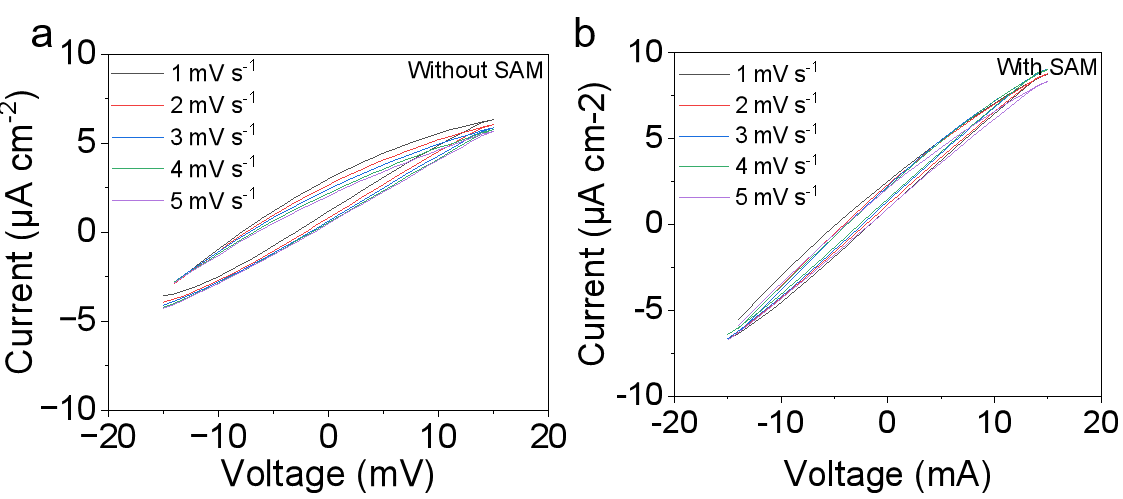


**Figure S11.** CV curves of Zn||Zn cells (a) without and (b) with SAM at different scan rates.


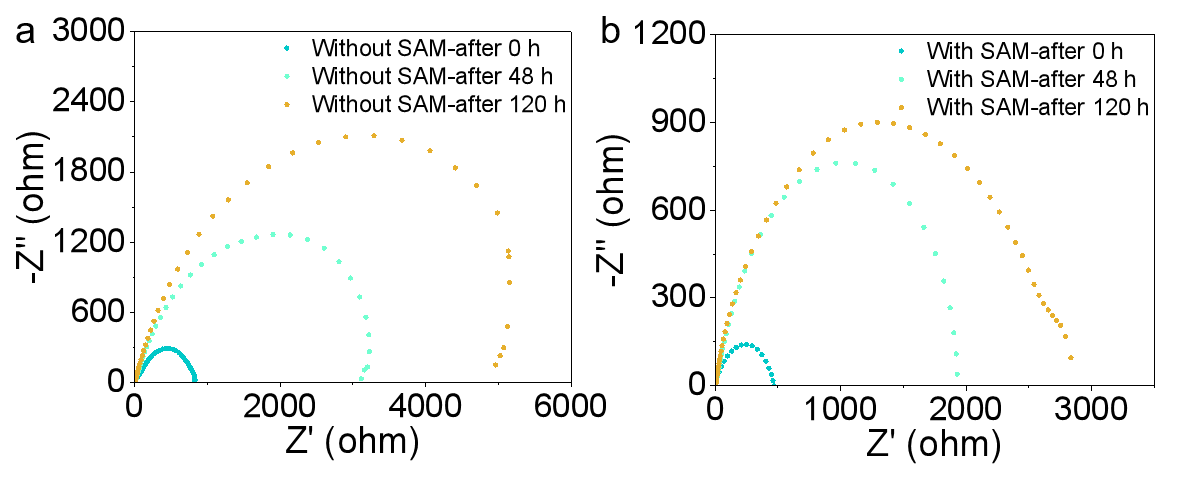


**Figure S12.** Electrochemical impedance spectroscopy of Zn||Zn cells (a) without and (b) with SAM after standing different times.


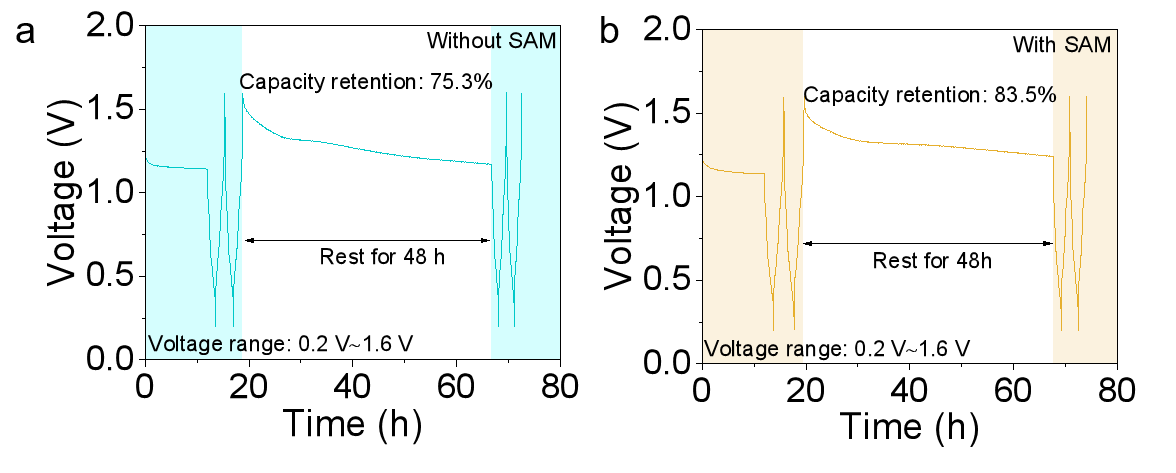


**Figure S13.** Self-discharge performance tests of NVO||Zn coin cells (a) without and (b) with SAM.


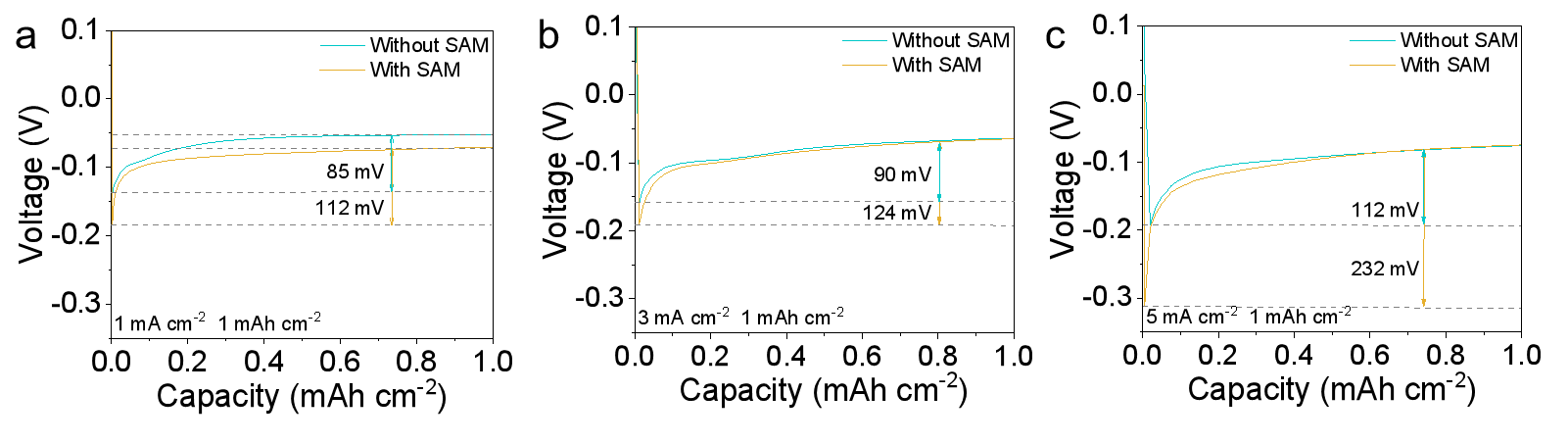


**Figure S14.** The first deposition voltage profiles of Zn||Cu cells at 1 mA cm^–2^/1 mAh cm^–2^, (b) 3 mA cm^–2^/1 mAh cm^–2^ and (c) 5 mA cm^–2^/1 mAh cm^–2^.


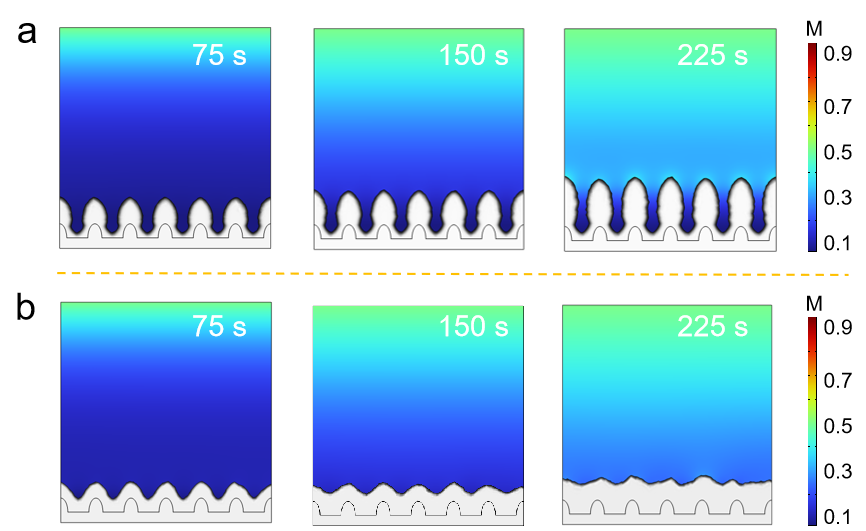


**Figure S15.** Simulated ion concentration distributions during the process of Zn deposition on the Zn anodes (a) without and (b) with SAM.


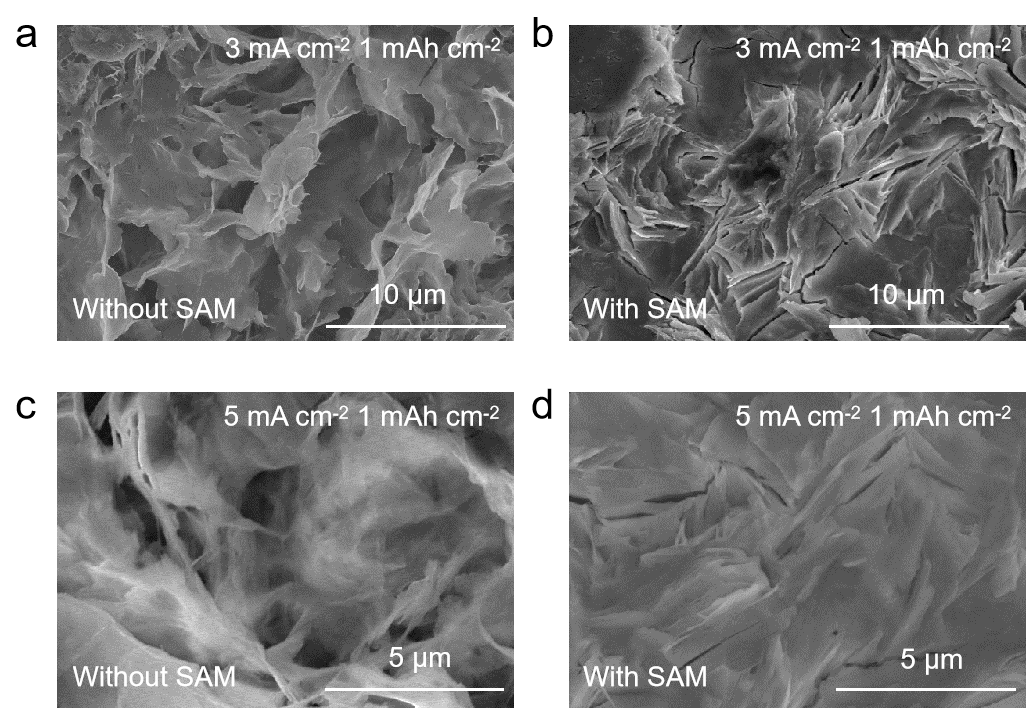


**Figure S16.** SEM images of Zn deposits with 1 mAh cm^−2^ capacity on anode surfaces (a, c) without and (b, d) with SAM at 3 and 5 mA cm^−2^.


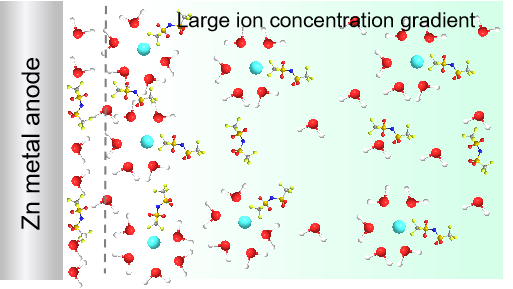


**Figure S17.** Schematic illustration of Zn electrodeposition without SAM.


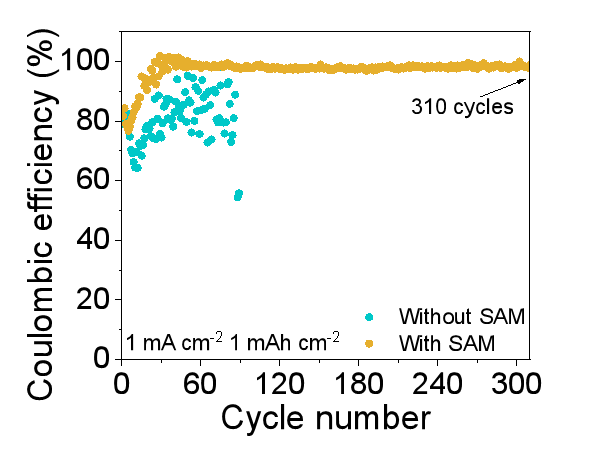


**Figure S18.** Coulombic efficiency of the Zn||Cu cells at 1 mA cm^–2^/1 mAh cm^–2^.


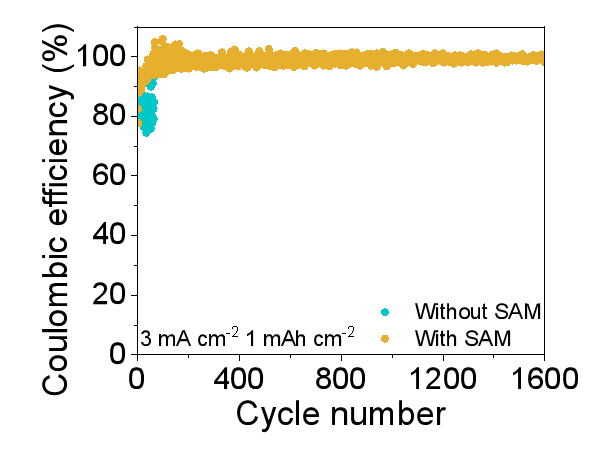


**Figure S19.** Coulombic efficiency of the Zn||Cu cells at 3 mA cm^–2^/1 mAh cm^–2^.


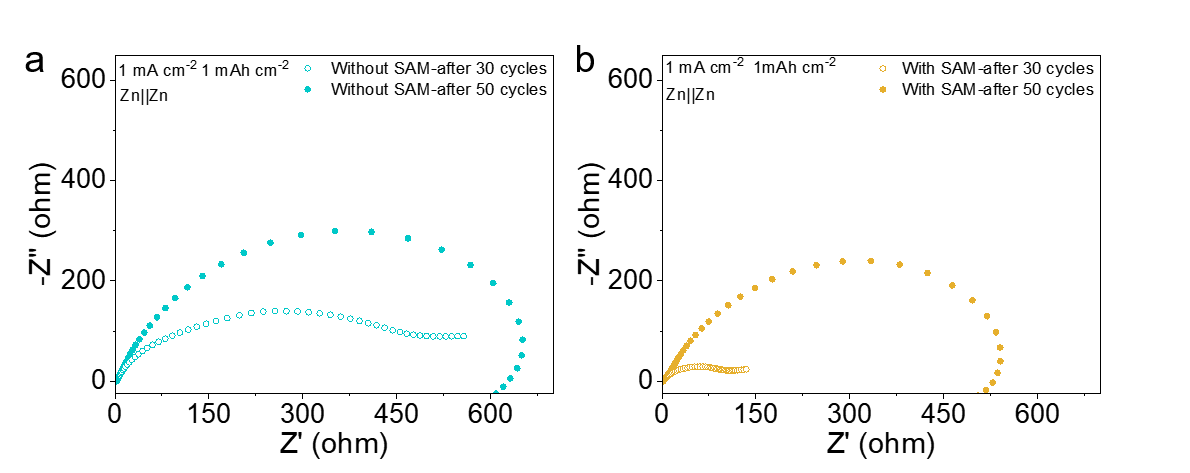


**Figure S20.** Electrochemical impedance spectroscopy of Zn||Zn cells (a) without and (b) with SAM after 30 and 50 cycles at 1 mA cm^–2^/1 mAh cm^–2^.


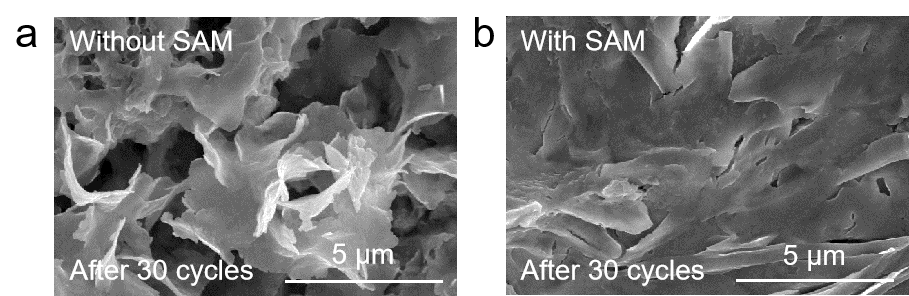


**Figure S21.** SEM images of Zn metal anodes (a) without and (b) with SAM after 30 cycles in Zn||Zn cells at 1 mA cm^–2^/1 mAh cm^–2^.


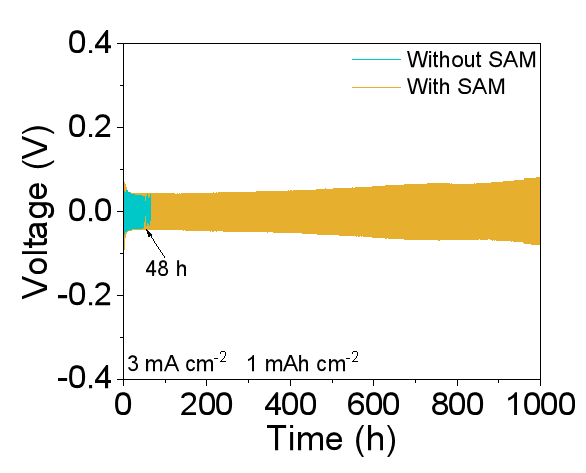


**Figure S22.** Cycling performance of Zn||Zn cells at 3 mA cm^–2^/1 mAh cm^–2^.


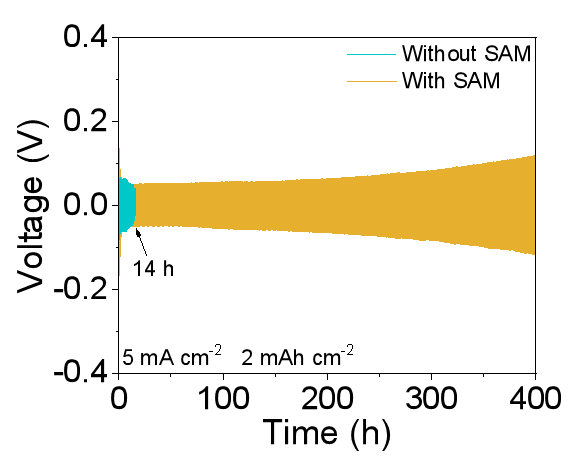


**Figure S23.** Cycling performance of Zn||Zn cells at 5 mA cm^–2^/2 mAh cm^–2^.


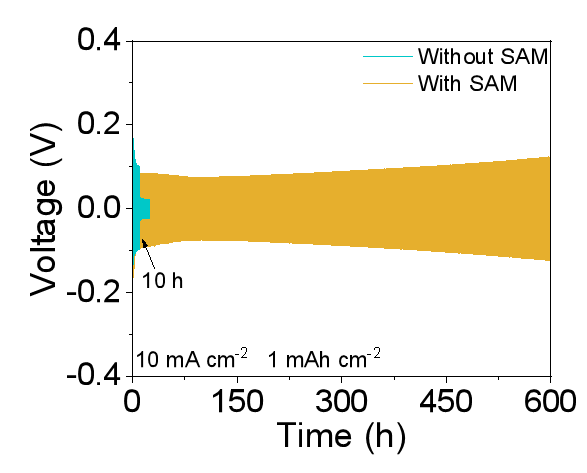


**Figure S24.** Cycling performance of Zn||Zn cells at 10 mA cm^–2^/1 mAh cm^–2^.


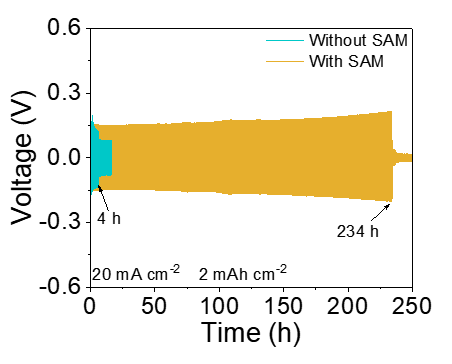


**Figure S25.** Cycling performance of Zn||Zn cells at 20 mA cm^–2^/2 mAh cm^–2^.


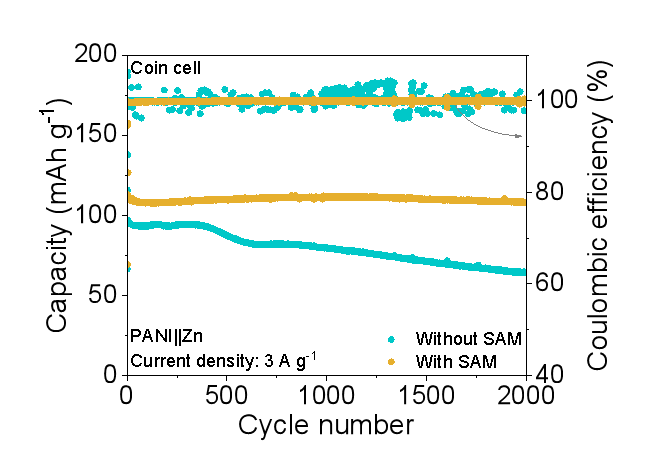


**Figure S26.** Cycling performance of PANI||Zn coin cells at 3 A g^−1^.


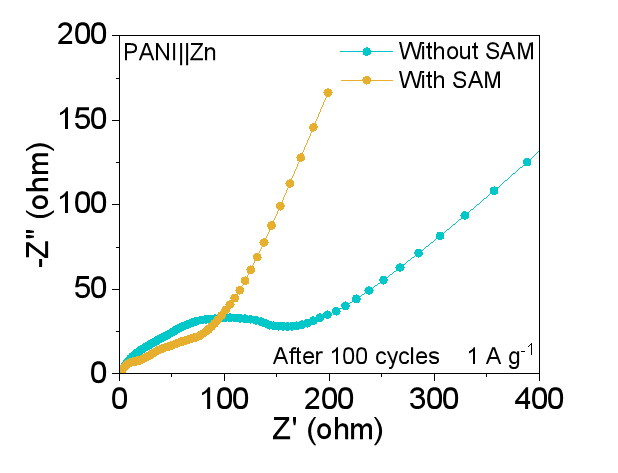


**Figure S27.** Electrochemical impedance spectroscopy of PANI||Zn coin cells after 100 cycles at 1 A g^−1^.


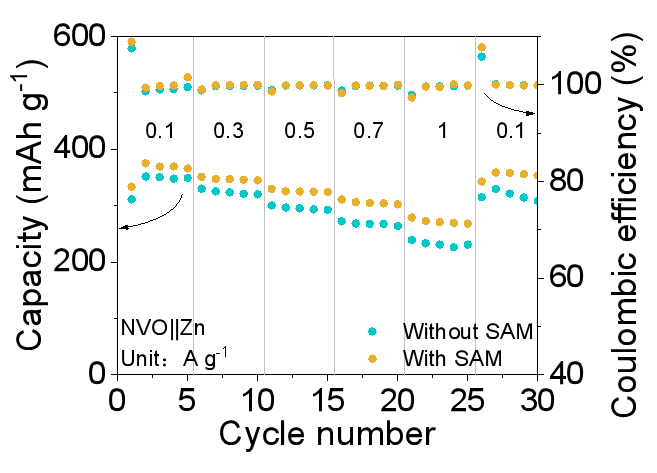


**Figure S28.** Rate performance of NVO||Zn coin cells.


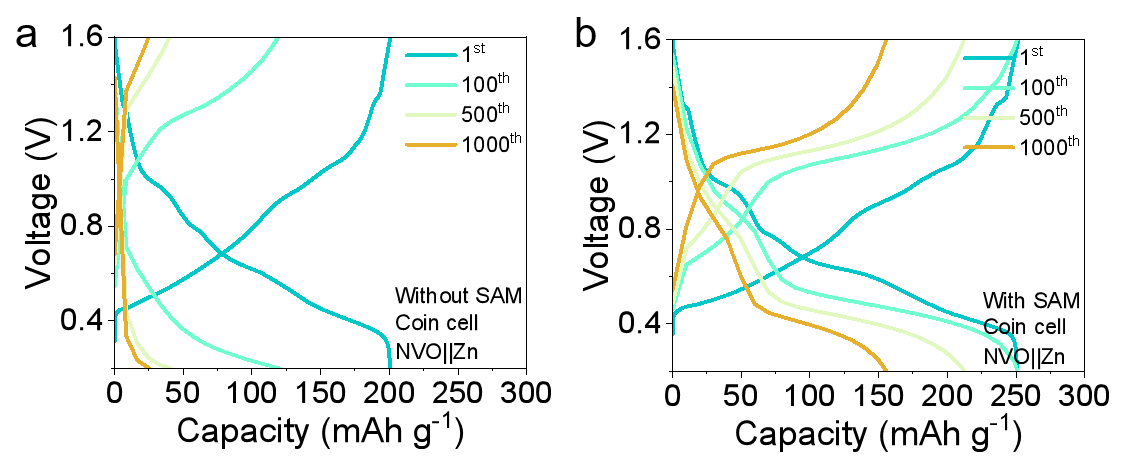


**Figure S29.** Typical charge/discharge curves of NVO||Zn coin cells (a) without and (b) with SAM at 3 A g^−1^.


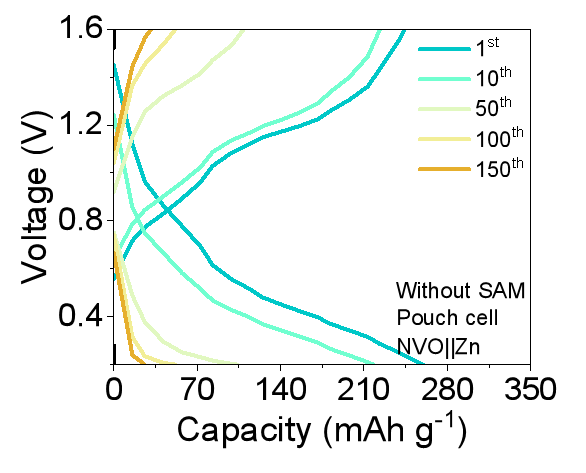


**Figure S30.** Typical charge/discharge curves of NVO||Zn pouch cells without SAM at 3 A g^−1^.

Table S1. Performance comparison between the SAM designed in this paper and the protective layer and electrolyte modification strategies reported in the literature.

|  | *Battery Types* | *Current density* | *Lifetime* | *Electrolyte* | *Reference* |
| --- | --- | --- | --- | --- | --- |
| *2* | *Zn\|\|Zn* | *1 mA cm^−2^*  *1 mAh cm^−2^* | *580 h* | *3 M ZnSO_4_* | *Nano Energy, 2022, 97, 107145.* |
| *3* | *Zn\|\|Zn* | *1 mA cm^−2^*  *1 mAh cm^−2^* | *750 h* | *1 M ZnSO_4_* | *ACS Nano, 2024, 18, 27672-27682.* |
| *4* | *Zn\|\|Zn* | *1 mA cm^−2^*  *1 mAh cm^−2^* | *900 h* | *2 M ZnSO_4_* | *Angew. Chem. Int. Ed. 2023, 62, e202301192.* |
| *5* | *Zn\|\|Zn* | *1 mA cm^−2^*  *0.5 mAh cm^−2^* | *1050 h* | *1 M ZnSO_4_* | *ACS Appl. Mater. Interfaces, 2024, 16, 30580-30588.* |
| *6* | *Zn\|\|Zn* | *1 mA cm^−2^*  *1 mAh cm^−2^* | *800 h* | *1 M ZnSO_4_* | *Chem. Eng. J. 2023, 452, 139557.* |
| *7* | *Zn\|\|Zn* | *1 mA cm^−2^*  *1 mAh cm^−2^* | *1000 h* | *2 M Zn(OTf)_2_* | *J. Colloid Interface Sci. 2023, 646, 950-958.* |
| *8* | *Zn\|\|Zn* | *1 mA cm^−2^*  *1 mAh cm^−2^* | *500 h* | *2 M ZnSO_4_* | *J. Colloid Interface Sci. 2022, 627, 367-374.* |
| *9* | *Zn\|\|Zn* | *1 mA cm^−2^*  *1 mAh cm^−2^* | *900 h* | *2 M ZnSO_4_* | *Angew. Chem. Int. Ed. 2023, 62, e202303557.* |
| *10* | *Zn\|\|Zn* | *1 mA cm^−2^*  *1 mAh cm^−2^* | *800 h* | *2 M ZnSO_4_* | *Adv. Funct. Mater. 2021, 31, 2104361.* |
| *11* | *Zn\|\|Zn* | *1 mA cm^−2^*  *1 mAh cm^−2^* | *400 h* | *2 M ZnSO_4_* | *Energy Storage Mater. 2021, 41 230-239.* |
| *12* | *Zn\|\|Zn* | *1 mA cm^−2^*  *0.5 mAh cm^−2^* | *360 h* | *2 M ZnSO_4_* | *ACS Appl. Energy Mater. 2022, 5, 2375-2383.* |
| *13* | *Zn\|\|Zn* | *1 mA cm^−2^*  *1 mAh cm^−2^* | *800 h* | *3 M ZnSO_4_* | *SusMat. 2024, 4, e189.* |
| *14* | *Zn\|\|Zn* | *1 mA cm^−2^*  *1 mAh cm^−2^* | *800 h* | *2 M ZnSO_4_* | *ACS Nano, 2022, 16, 6906-6915.* |
| *15* | *Zn\|\|Zn* | *1 mA cm^−2^*  *1 mAh cm^−2^* | *1096 h* | *2 M Zn(TFSI)_2_* | *J. Power Sources, 2023, 570, 233048.* |
| *16* | *Zn\|\|Zn* | *1 mA cm^−2^*  *1 mAh cm^−2^* | *1000 h* | *2 M ZnSO_4_* | *Chem. Eng. J. 2023, 464, 142580.* |
|  | *Zn\|\|Zn* | *1 mA cm^−2^*  *1 mAh cm^−2^* | *1400 h* | *1 M Zn(TFSI)_2_* | *This work* |

1. J. Sun, J. Zhang, S. Wang, P. Sun, J. Chen, Y. Du, S. Wang, S. Ismael, Y. Wang and Y. Wei, *Energy Environ. Sci*. 2024, **17**, 4304-4318.
2. Z. Miao, Q. Liu, W. Wei, X. Zhao, M. Du, H. Li, F. Zhang, M. Hao, Z. Cui, Y. Sang, X. Wang, H. Liu, S. Wang, *Nano Energy*, 2022, **97**, 107145.
3. K. Wang, Y. Luo, H. Zhan, X.-X. Liu, X. Sun, *ACS Nano*, 2024, **18**, 27672-27682.
4. Y. Dai, C. Zhang, W. Zhang, L. Cui, C. Ye, X. Hong, J. Li, R. Chen, W. Zong, X. Gao, J. Zhu, P. Jiang, Q. An, D. J. L. Brett, I. P. Parkin, G. He, L. Mai, *Angew. Chem. Int. Ed*. 2023, **62**, e202301192.
5. W. Chen, Z. Xie, H. Chen, X. Wang, *ACS Appl. Mater. Interfaces*, 2024, **16**, 30580-30588.
6. H. Peng, X. Wang, F. Yang, Z. Liu, H. Lei, S. Cui, X. Xie, G. Ma, *Chem. Eng. J*. 2023, **474**, 145864.
7. R. Wang, L. Liu, S. Huang, Y. Wu, X. Chen, Z. Liang, J. Xu, *J. Colloid Interface Sci.* 2023, **646**, 950-958.
8. H. Cao, X. Huang, Y. Liu, Q. Hu, Q. Zheng, Y. Huo, F. Xie, J. Zhao, D. Lin, *J. Colloid Interface Sci*. 2022, **627**, 367-374.
9. R. Sun, D. Han, C. Cui, Z. Han, X. Guo, B. Zhang, Y. Guo, Y. Liu, Z. Weng, Q.-H. Yang, *Angew. Chem. Int. Ed*. 2023, **62**, e202303557.
10. S. Zhou, Y. Wang, H. Lu, Y. Zhang, C. Fu, I. Usman, Z. Liu, M. Feng, G. Fang, X. Cao, S. Liang, A. Pan, *Adv. Funct. Mater*. 2021, **31**, 2104361.
11. Y. Yang, C. Liu, Z. Lv, H. Yang, X. Cheng, S. Zhang, M. Ye, Y. Zhang, L. Chen, J. Zhao, C. C. Li, *Energy Storage Mater.* 2021, **41** 230-239.
12. Y. Wang, H. Sun, N. Li, K. Chen, X. Yang, H. Liu, G. Zheng, J. Liu, Z. Wu, L. Zhai, L. Mi, *ACS Appl. Energy Mater.* 2022, **5**, 2375-2383.
13. J. Dong, J. Duan, R. Cao, W. Zhang, K. Fang, H. Yang, Y. Liu, Z. Shen, F. Li, R. Liu, M. Jin, L. Lei, H. Li, C. Chen, *SusMat*. 2024, **4**, e189.
14. L. Hong, X. Wu, L.-Y. Wang, M. Zhong, P. Zhang, L. Jiang, W. Huang, Y. Wang, K.-X. Wang, J.-S. Chen, *ACS Nano*, 2022, **16**, 6906-6915.
15. B. Huang, J. Song, H. Kimura, Y. Li, Y. Xu, K. Yang, M. Cui, L. Du, L. Kang, *J. Power Sources*, 2023, **570**, 233048.
16. Y.-H. Lee, Y. Jeoun, S.-H. Lee, J. H. Kim, S.-Y. Kim, S.-H. Yu, K.-S. Ahn, Y.-E. Sung, *Chem. Eng. J.* 2023, **464**, 142580.
